# Assessment of Empirical Troposphere Model GPT3 Based on NGL’s Global Troposphere Products

**DOI:** 10.3390/s20133631

**Published:** 2020-06-28

**Authors:** Junsheng Ding, Junping Chen

**Affiliations:** 1 Shanghai Astronomical Observatory, Chinese Academy of Sciences, Shanghai 200030, China; dingjunsheng@shao.ac.cn; 2 School of Astronomy and Space Science, University of Chinese Academy of Sciences, Beijing 100049, China; 3 Shanghai Key Laboratory of Space Navigation and Positioning Techniques, Shanghai 200030, China

**Keywords:** GPT3 troposphere model, NGL products, ZTD, gradient, GNSS

## Abstract

Tropospheric delay is one of the major error sources in GNSS (Global Navigation Satellite Systems) positioning. Over the years, many approaches have been devised which aim at accurately modeling tropospheric delays, so-called troposphere models. Using the troposphere data of over 16,000 global stations in the last 10 years, as calculated by the Nevada Geodetic Laboratory (NGL), this paper evaluates the performance of the empirical troposphere model GPT3, which is the latest version of the GPT (Global Pressure and Temperature) series model. Owing to the large station number, long time-span and diverse station distribution, the spatiotemporal properties of the empirical model were analyzed using the average deviation (BIAS) and root mean square (RMS) error as indicators. The experimental results demonstrate that: (1) the troposphere products of NGL have the same accuracy as the IGS (International GNSS Service) products and can be used as a reference for evaluating general troposphere models. (2) The global average BIAS of the ZTD (zenith total delay) estimated by GPT3 is −0.99 cm and the global average RMS is 4.41 cm. The accuracy of the model is strongly correlated with latitude and ellipsoidal height, showing obviously seasonal variations. (3) The global average RMS of the north gradient and east gradient estimated by GPT3 is 0.77 mm and 0.73 mm, respectively, which are strongly correlated with each other, with values increasing from the equator to lower latitudes and decreasing from lower to higher latitudes.

## 1. Introduction

The Global Navigation Satellite Systems (GNSS) signals are delayed and bent as they pass through the atmosphere, and the positioning error due to this is defined as atmospheric delay. The portion of the atmospheric delay caused by the ionosphere is called the ionospheric delay, while the portion caused by the unionized neutral atmosphere is called the tropospheric delay. The tropospheric delay can be divided into hydrostatic delay (HD) and wet delay (WD) [1,2]. The hydrostatic delay accounts for 90% of the total tropospheric delay and can be accurately estimated by the model with a calculation accuracy of up to millimeters [3]. Although the percentage of wet delay is small, it is arduous to model because of its strong correlation with water vapor content, which varies dramatically from time to time and place to place [4,5]. The wet delay is the main limiting factor of the tropospheric delay modeling accuracy, which is often involved in calculations as unknown parameters in precision positioning.

The general approach of model estimation is to construct an empirical tropospheric zenith total delay (ZTD) model, which is then corrected to the direction of signal propagation by a mapping function (MF). Consequently, the accuracy of the ZTD calculated by the model directly affects the accuracy of the tropospheric delay. The commonly used tropospheric models can be divided into three categories according to the source of modeling data. The first category is the tropospheric model based on radiosonde observations, such as the Hopfield model [6] and the Saastamoinen model [7]. Such models have underdeveloped real-time performance due to the need to measure meteorological data as input. The second category is a tropospheric model based on discrete ZTD data, such as the global tropospheric model established in paper [8] and SHAtropE [9] established by the Shanghai Astronomical Observatory GNSS Analysis Centre. Such models are independent of meteorological parameters, easy to use, and have outstanding accuracy, but they require ZTD data over a long-time span and have the highest modeling costs of any model. The third category is a tropospheric model based on standard atmospheric or reanalyzed data, such as the UNB (University of New Brunswick) series model based on the US standard atmosphere [10] and GPT (Global Pressure and Temperature) series model based on the reanalysis of numerical weather model (NWM) products provided by the European Centre for Medium-Range Weather Forecasts (ECMWF) [11,12,13,14]. These models are easy to use, accurate, and widely used.

The UNB series model was originally established to estimate the meteorological parameters required for the US Wide Area Augmentation System (WAAS) [15], followed by a series of improved versions. Currently, the UNB series models include UNB1-4, UNB3m, and UNB.na [16]. Based on NWM products, the GPT series models provide meteorological parameters such as temperature, pressure, and water vapor pressure at any position on the Earth’s surface. At present, there are 4 versions: GPT, GPT2, GPT2w, and GPT3. The accuracy of GPT2 as evaluated using GNSS data in China is 4.65 cm [17]. GPT2w adds two parameters of temperature lapse rate and mean temperature of the water vapor based on GPT2, considered to be the most accurate tropospheric model for quite a long time. GPT3 is for an upgraded version of GPT2w, developed along with VMF3 (Vienna Mapping Functions 3), which is based on ERA-Interim Pressure-Level Data, compared with GPT2w, two parameters of gradients in the north direction (Grad.N) and the east direction (Grad.E) are added [14].

Since the GPT3 model was made available, no relevant literature has emerged to effectively assess the accuracy of its calculated ZTD. Based on this, this paper uses more than a decade of tropospheric data published by the Nevada Geodetic Laboratory from more than 16,000 stations worldwide [18], to access the GPT3 model using average deviation and root mean square error as accuracy metrics and characterizes the distribution of model accuracy in time and space. It should be noted that the GPT series models do not directly provide ZTD, but rather the meteorological parameters required by the first category of the model, which is then used to calculate ZTD, in other words, the ZTD evaluated in this study is calculated by GPT3+Saastamoinen.

The structure of this paper is as follows: in Section 1, the current development of the tropospheric model and the current state of the GPT series models are introduced. Section 2 describes the assessment materials and methods. In Section 3, the assessment results are presented and the temporal and spatial distribution characteristics of GPT3 model are analyzed. Section 4 gives the conclusions.

## 2. Materials and Methods

This study focuses on the assessment of the GPT3 model using America Nevada Geodetic Laboratory (NGL) troposphere products, to begin with, we provide a brief background on the materials and assessment methods used. In this section, the development of GPT series models and NGL products are briefly introduced; then the accuracy of NGL products is evaluated and the feasibility of using NGL products to assess the tropospheric model is analyzed; finally, the assessment methods used in this article are described.

### 2.1. Development of Global Pressure and Temperature (GPT) Series Model

The empirical model GPT, which is based on spherical harmonics up to degree and order nine, uses the monthly average grid data ERA40 of 40 years of global temperature and pressure with a spatial resolution of 15° × 15° provided by ECMWF, provides pressure and temperature at any site in the vicinity of the Earth’s surface [11]. Due to the limited spatial and temporal variability of GPT, Lagler et al. (2013) provide GPT2, which provides not only higher accuracy pressure and temperature, but also temperature lapse rate, water vapor pressure, and mapping function coefficients [12]. After this, the water vapor decrease factor and the mean temperature of the water vapor were added to the new version: GPT2w [13]. As the latest version, GPT3 is developed along with the VMF3 mapping function, which contains hydrostatic and wet empirical mapping function coefficients derived from special averaging techniques of the respective (discrete) VMF3 data, and its meteorological quantities are adopted as the stands from GPT2w. In addition, the north gradient and east gradient were added to the output parameters of GPT3 [14,19].

### 2.2. Nevada Geodetic Laboratory (NGL) Troposphere Products

Until 5 November 2017, NGL provided over 34 million station-days of troposphere products (total zenith delay, north gradient, and east gradient) every 5 min since 1996 from over 16,000 stations. As of November 2019, these data were updated to 43 million, 1994, 18,000, and the number of stations is still increasing at a rate of about 1000 per year. In this update, products are generated using more advanced modeling and data processing, where integrated water vapor (at zenith) and weighted mean tropospheric temperature are added to the products for the first time. The NGL products are generated using JPL’s GipsyX 1.0 software with JPL’s Repro 3.0 orbits and clocks, VMF1 gridded data and mapping function parameters as inputs. Its format follows the IGS SINEX_TRO standard and they are available at http://geodesy.unr.edu/gps_timeseries/trop/.

**Figure 1:**
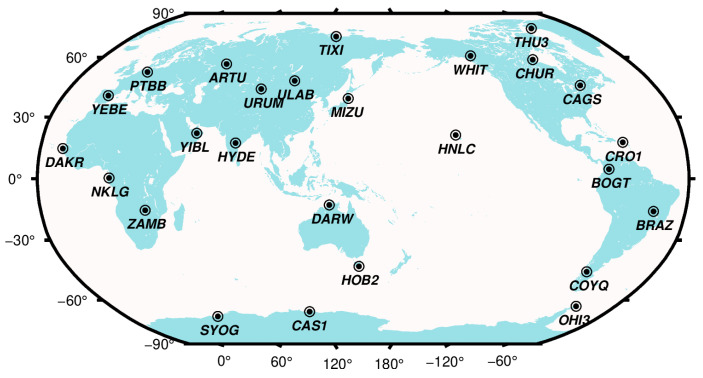
Global distribution of 26 selected IGS and NGL common stations.

Thanks to its large number of more than 16,000 stations, NGL troposphere products provide ideal data sources for the evaluation of empirical tropospheric models, which could derive ZTDs at any point on the ground. However, the accuracy of NGL troposphere products needs to be evaluated. To judge whether the NGL troposphere products have sufficient accuracy to evaluate the empirical troposphere models, 26 global IGS and NGL common stations are selected to evaluate the accuracy of NGL products, with a data span from January 2009 to April 2019, these selected stations are depicted in Figure 1. To ensure that these selected samples are representative, the following principles are observed when selecting stations: (1) The global distribution of stations is ensured to be roughly uniform, mainly in terms of longitude and latitude. (2) Stations in special areas such as bipolar regions and oceanic regions (stations in islands) are given consideration. (3) Ensure enough stations in the inland area (since the distribution of stations is highly correlated with the location of the city).

In this paper, the average deviation (BIAS) and root mean square (RMS) error are used as the accuracy indicators. The calculation methods of BIAS and RMS can be expressed as:

(1) $$\{\begin{array}{l} \mathrm{BIAS}=\frac{1}{N}\sum_{i=1}^{N}\left({\mathrm{ZTD}}_{i}^{\mathrm{NGL}}-{\mathrm{ZTD}}_{i}^{\mathrm{IGS}}\right)\\ \mathrm{RMS}=\sqrt{\frac{1}{N}\sum_{i=1}^{N}{\left({\mathrm{ZTD}}_{i}^{\mathrm{NGL}}-{\mathrm{ZTD}}_{i}^{\mathrm{IGS}}\right)}^{2}} \end{array}$$

where N denotes the sampling number of the ZTD times series, ${\mathrm{ZTD}}_{i}^{\mathrm{NGL}}$ and ${\mathrm{ZTD}}_{i}^{\mathrm{IGS}}$ are the ZTD at the sample epoch of NGL ZTD and IGS, respectively. The time resolution of the ZTD times series in IGS and products is 5 min. In this experiment, it is resampled to 1 h.

**Figure 2:**
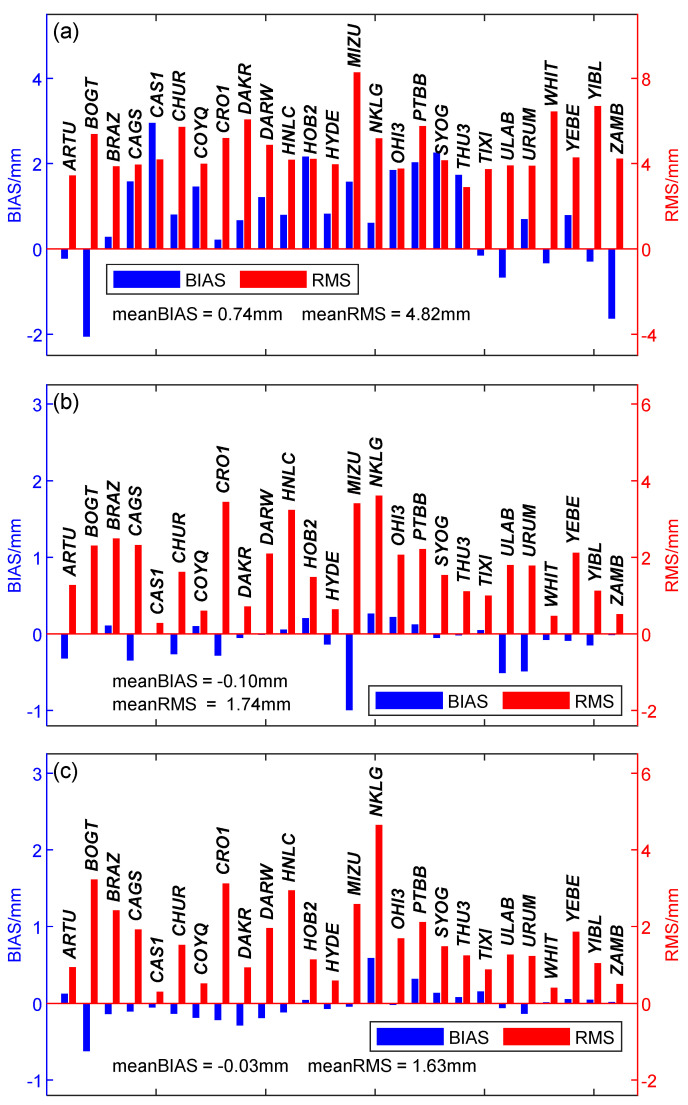
Average deviation (BIAS) (blue) and root mean square (RMS) error (red) of NGL zenith total delay (ZTD) (**a**), gradients in the north direction (Grad.N) (**b**), and gradients in the east direction (Grad.E) (**c**), with data span from January 2009, to April 2019.

The statistical results are displayed in Figure 2, which shows the BIAS and RMS of each station. It can be found from the Figure 2 that the variation range of BIAS and RMS between the stations is relatively small, and the BIAS value of most stations is kept within the range of ±2 mm, while the RMS value is shown to fluctuate around 4 mm. The ZTD average BIAS and RMS of 26 stations selected are 0.74 mm and 4.82 mm, the Grad.N average BIAS and RMS are −0.10 mm and 1.74 mm, and the Grad.E average BIAS and RMS are −0.03 mm and 1.63 mm. The values are close to the official nominal accuracy of IGS ZTD (the accuracy of IGS ZTD is 4 mm, http://www.igs.org/products), and it can be concluded that NGL ZTD has the same accuracy as IGS ZTD and can be used to assess empirical tropospheric models.

On proving that the NGL products have the same accuracy as the IGS products, the NGL products are used as an accuracy reference to assess the GPT3 model. Using the GPT3 model, parameters such as ZTD were calculated for more than 16,000 NGL stations over a 10-year period, and then the BIAS and RMS values for each of these parameters were calculated with reference to Equation (1).

## 3. Results and Analysis

This section presents the results of the assessment of GPT3 and characterizes the accuracy distribution of the three parameters (zenith tropospheric delay, ZTD; the northern gradient, Grad.N; the eastern gradient, Grad.E) calculated using the model in terms of temporal and spatial directions, respectively.

### 3.1. Time Distribution Characterization of Model Accuracy

**Figure 3:**
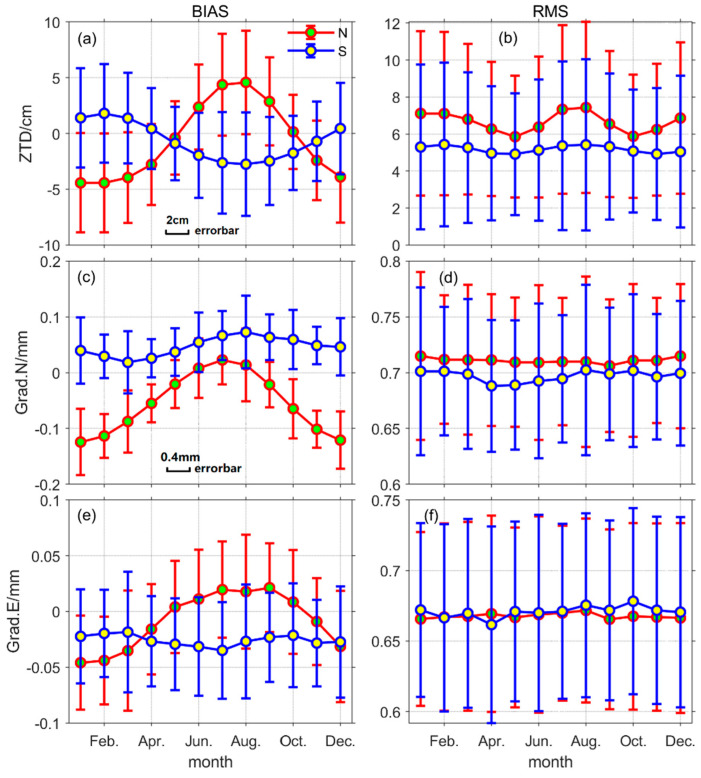
Average monthly BIAS (**a**,**c**,**e**) and RMS (**b**,**d**,**f**) values of zenith troposphere delay derived from GPT3 (Global Pressure and Temperature series model), north gradient and east gradient in the northern (green) and southern (yellow) hemispheres. The error bar stands for the standard deviation (STD) of each month. (Note: (**a**,**b**) share the error bar scale, (**c**–**f**) share the error bar scale).

To obtain the time distribution characteristics of the model accuracy, the BIAS, RMS, and standard deviation (STD) value of ZTD, Grad.N, and Grad.E from more than 16,000 stations are divided into 12 groups according to the observation months, due to the difference between the northern and southern hemispheres, each group is also divided into two subgroups: south and north. Next, the mean and standard deviation of BIAS and RMS for each parameter in each subgroup were calculated. Finally, the statistical results are depicted in Figure 3.

In Figure 3, N and S denote the northern and southern hemispheres, respectively, where subgraph (a) and subgraph (b) depict the monthly average of BIAS and RMS values of GPT3 ZTD, respectively. The information of distribution characteristics expressed in these two subgraphs can be summarized as follows: (1) BIAS values exhibit sinusoidal fluctuations with an annual cycle and shows opposite signs for regions in the northern and southern hemispheres, where the northern hemisphere wave peaks occur in August and troughs in February. Amplitude is of about 5 cm and 2 cm in the northern and southern hemispheres, respectively; the mean value of the southern hemisphere data is not as close to zero as it is in the northern hemisphere, but at about −1 cm. (2) The RMS values also appear as a sine wave, but the cycle becomes semi-annual and the northern and southern hemispheres become congruent, with the two peaks occurring in February and August, respectively; the amplitude of the northern hemisphere is about 1 cm, with an average value of about 6.5 cm, while the southern hemisphere is almost zero, with an average value of about 5.5 cm. (3) The STD of both BIAS RMS is proportional to their absolute value, indicating that the model is more reliable for months with higher accuracy than for months with lower accuracy.

The subgraphs (c)–(f) in Figure 3 are the average monthly BIAS and RMS values of Grad.N and Grad.E, respectively. By analyzing these subgraphs, it can be summarized as the following points: (1) The fluctuating trends in the BIAS of Grad.N and Grad.E are consistent with the BIAS of ZTD with the same phase in the northern and southern hemispheres; the BIAS of Grad.N and Grad.E has much smaller amplitudes in the southern hemisphere than in the northern hemisphere, and in particular, the sinusoidal trend of Grad.E BIAS in the southern hemisphere is almost non-visible; the mean of BIAS of Grad.N in the southern hemisphere is greater than zero, and the mean of Grad.N BIAS in the northern hemisphere is less than zero, while the mean of BIAS of Grad.E in both the southern and northern hemispheres is less than zero. (2) The RMS of Grad.N and Grad.E fluctuated little and did not differ significantly in the northern and southern hemispheres; the mean value of Grad.E RMS is smaller than Grad.N. (3) The STD of BIAS in Grad.N and Grad.E was the same as the STD regularity of ZTD BIAS, whereas no significant regularity was found in the STD of RMS in Grad.N and Grad.E.

### 3.2. Spatial Distribution Characterization of Model Accuracy

**Figure 4:**
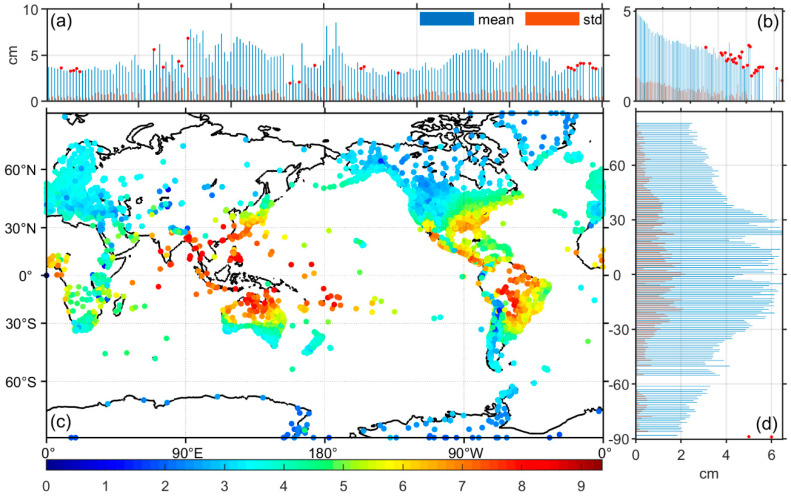
Root mean square (RMS) values calculated by GPT3 troposphere model at various stations around the world (**c**), and the relationship between RMS value and longitude (**a**), ellipsoid height (**b**) and latitude (**d**). The RMS mean values (blue) and standard deviation (std, orange) within 1 degree of latitude/longitude or 50 m of ellipsoid height are made into a histogram, the small red dot indicates positive BIAS value. (Note: the vertical coordinates in (**b**) represent RMS values, as in (**a**), and the horizontal coordinates represent the grouping number of the station’s ellipsoid height from small to large, followed by a 50 m interval).

**Figure 5:**
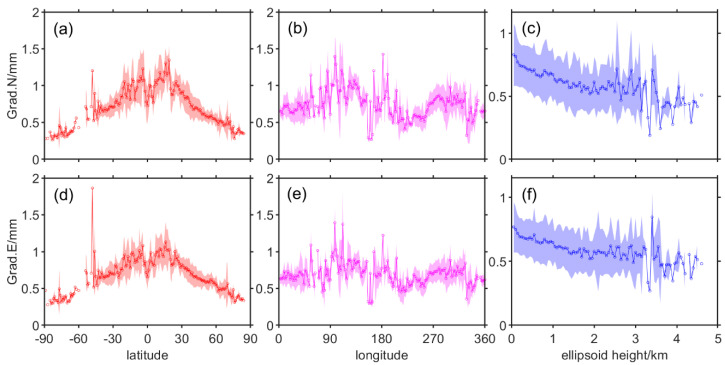
The relationship between Grad.N and Grad.E root mean square (RMS) values with longitude (**b**,**e**), latitude (**a**,**d**) and ellipsoid height (**c**,**f**).

The data processing of the spatially distributed characteristics of the model accuracy is a little more complicated than that of the temporal distribution, as the three aspects of longitude, latitude, and ellipsoid height are discussed spatially, respectively. BIAS and RMS data from more than 16,000 stations were grouped at 1-degree intervals at longitude and latitude, respectively, and every 50 m at ellipsoid height. The statistical results for the ZTD data are depicted in Figure 4, while the results for Grad.N and Grad.E are depicted in Figure 5.

Subgraph (c) of Figure 4 provides the location of each station and their mean RMS of ZTD, with the color bar representing the magnitude of the mean value. From subgraph (c), it can be seen that the RMS has significant geographical features, so is the distribution of the ZTD RMS in terms of longitude, ellipsoid height and latitude is further shown in subgraph (a), (b) and (d), respectively. Considering that the absolute and RMS values of BIAS have similar fluctuating trends, BIAS is not depicted but is illustrated by the red dotted marker for positive mean BIAS value in each subgroup. The information expressed in Figure 4 can be synthesized into the following: (1) the RMS values of ZTD calculated using GPT3 is not significantly correlated with the longitude of the station site; the overall positive and negative BIAS values are independent of longitude, but there are small positive BIAS aggregations in the 0 to 30 degrees west longitude region. (2) The RMS values are negatively correlated with the ellipsoid height, i.e., the GPT3 model is more accurate in regions with higher ellipsoid heights; BIAS values are mostly positive in regions where the ellipsoid height is greater than 3000 m and almost always negative in regions less than 3000 m. This means that the ZTD calculated by the GPT3 model is underestimated below 3000 m and overestimated above 3000 m. (3) The accuracy of ZTD calculated by GPT3 is strongly correlated with the latitude of the station, and the accuracy of ZTD at high latitudes is higher than at low latitudes; BIAS values are almost exclusively negative in all latitude groups. (4) The magnitude of the STD value is positively correlated with the RMS value, independent of latitude and longitude and ellipsoid height.

The six subgraphs of Figure 5 demonstrate the relationship between the accuracy of Grad.N and Grad.E with longitude, latitude, and ellipsoid height, respectively. In these subgraphs, the small circles represent the mean value of the RMS in each subgroup, and the translucent regions are its scopes represented by the STD. Since Grad.N and Grad.E perform similarly in each spatial dimension, the analysis is presented here from each of these three dimensions. The first is latitude, RMS values are negatively correlated with latitude, but become positively correlated in the equatorial region, it shows the “M” shape of the twin peaks, with the peak of the twin peaks roughly at the latitude of the north–south regression line. This may be related to the fact that these two regions are subtropical high-pressure zones. The second is longitude, the magnitude of RMS values do not correlate significantly with longitude, but smaller values can be seen in the Pacific region. The third is ellipsoid height, the RMS value decreases with increasing ellipsoid height and its shape approximates the logarithmic function because the rate of decline with height decreases, and the whole curve becomes closer and closer to the constant. Finally, STD varies little across all types of subgroups and is only somewhat larger in the low latitude region, the longitude region where the Americas are located. The lower latitudes have high moisture content due to high temperatures, while the American region may be due to Amazonian forests.

Synthesizing Figure 3, Figure 4 and Figure 5, it is easy to find an association between ZTD and gradient. The correlation coefficients between ZTD, Grad.N and Grad.E are calculated, and the results are shown in Table 1. From the Table 1, it can be found that a strong correlation exists between the RMS of Grad.N and Grad.E with a correlation coefficient of 0.8029 between them, and this value is 0.6581 and 0.5662 between ZTD RMS and Grad.N RMS and Grad.E RMS, respectively.

## 4. Conclusions

In this paper, we describe the development of GPT series models and the NGL troposphere products with more than 10,000 stations worldwide over a 20-year span and, then, the NGL troposphere products are evaluated using IGS troposphere products as true values, and the results show that NGL products have the same accuracy as IGS. On this basis, over 10 years of NGL data from more than 16,000 stations worldwide were used to evaluate the GPT3 model. The accuracy of the three parameters (zenith tropospheric delay, ZTD; the northern gradient, Grad.N; the eastern gradient, Grad.E) calculated using the GPT3 model was evaluated and analyzed in a total of four dimensions in time and space (longitude, latitude, and ellipsoid height), and the following conclusions have been drawn:

(1) The global average BIAS of ZTD, Grad.N, and Grad.E calculated by GPT3 is −0.99 cm, −0.029 mm, −0.016 mm, respectively, and the global average RMS is 4.41 cm, 0.77 mm, 0.73 mm, respectively. The BIAS and RMS values for all three parameters exhibit spatiotemporal distribution characteristics.

(2) The BIAS of ZTD, Grad.N, and Grad.E and the RMS of ZTD all show obvious seasonal variations, with the BIAS of ZTD in the opposite phase of the northern and southern hemispheres. The magnitude of this seasonal change is smaller in the southern hemisphere than in the northern hemisphere.

(3) The RMS of ZTD, Grad.N, and Grad.E are negatively correlated with the ellipsoidal height and latitude while not significantly correlated with longitude. The RMS of Grad.N and Grad.E shows an “M” shape in relation to latitude, which may be related to the subtropical high-pressure zone. In addition, there is a strong correlation between the RMS of Grad.N and Grad.E.

## Figures and Tables

**Table 1:** Correlation coefficient.

	ZTD	Grad.N	Grad.E
ZTD	1	0.6581	0.5662
Grad.N	0.6581	1	0.8029
Grad.E	0.5662	0.8029	1
